# Atypical lobular endocervical glandular hyperplasia: two case report and literature review

**DOI:** 10.3389/fonc.2023.1298793

**Published:** 2023-12-05

**Authors:** Ziqing Wan, Shuang Liu, Na Sang, Yi Tang, Peng Wen, Pu Zhang, Chuqiang Shu

**Affiliations:** Department of Obstetrics & Gynecology, Hunan Provincial Maternal and Child Health Care Hospital, Changsha, China

**Keywords:** atypical lobular endocervical glandular hyperplasia, gastric-type adenocarcinoma, human papillomavirus, pathology, diagnosis, therapy

## Abstract

Atypical lobular endocervical glandular hyperplasia (ALEGH) is considered a precancerous lesion of gastric-type adenocarcinoma (GAS)/minimal deviation adenocarcinoma (MDA) characterized by an insidious onset, atypical symptoms, and often negative human papillomavirus (HPV) screening. Early screening for this disease is challenging, leading to a high rate of missed clinical diagnoses and the development of malignant tumors at the onset. Increased vaginal discharge and the presence of imaging cystic masses at the internal cervical ostium are often observed in patients with ALEGH. Therefore, we reviewed the clinical data of two cases of ALEGH that were identified and diagnosed in the early stages at our hospital. Through a comprehensive analysis of the medical history and diagnosis plan, combined with a review of relevant literature, to improve the early recognition and diagnosis of ALEGH, as well as strengthen the management of cervical precancerous lesions.

## 1 Introduction

Lobular endocervical glandular hyperplasia (LEGH) is a rare proliferation of the cervical glands, first reported by Nucci et al. in 1999 (1). An atypical form of LEGH, known as atypical lobular endocervical glandular hyperplasia (ALEGH), exhibits architectural and cytological abnormalities, including enlarged nuclei, irregular nuclear membranes, prominent nucleoli, coarse chromatin, apoptotic bodies, and mitotic figures, in accordance with the characteristics of LEGH, and the glandular epithelium may display folding, clustering, or papillary expansion (2). Recent research suggests that ALEGH may serve as a potential precursor to gastric-type adenocarcinoma (GAS)/minimal deviation adenocarcinoma (MDA), both of which have been associated with a poor prognosis (3–5). However, as ALEGH often presents with subtle symptoms and yields negative results in human papillomavirus (HPV) screening, it easily overlooked in clinical practice and leading to disease progression. To address this issue, we present two cases of promptly identified ALEGH and conduct a retrospective examination of relevant research on LEGH/ALEGH by exploring databases such as PubMed and ClinicalKey, aiming to summarize the clinical and pathological characteristics of LEGH/ALEGH and provide insights into its diagnosis and treatment.

## 2 Case report

The first case we report is a 47-year-old female. The patient complained of vaginal discharge for the past 5 years. HPV screening and thinprep cytologic test (TCT) conducted at our clinic did not reveal any significant abnormalities. In the past, the patient underwent modified radical surgery for intermediate-grade ductal carcinoma *in situ* of the right breast in 2017. Postoperatively, she received endocrine therapy with daily oral administration of tamoxifen (60mg) and regular follow-up examinations showed no signs of recurrence. The patient did not undergo regular cervical cancer screening or receive HPV vaccination. She has had one full-term delivery, one cesarean section, one induced abortion, and two miscarriages. There is no family history of tumors or genetic diseases.

Upon admission, a gynecological examination was performed. The vagina showed a moderate amount of clear and watery discharge, and the surface of the cervix appeared smooth without any signs lesion. The cervix was enlarged with a diameter of approximately 4cm and had a slightly firm texture, without involvement of the parametrium. The ultrasound examination indicates a uterine endometrial thickness of 1.29cm, with no reported significant cervical mass.

Considering the patient’s history of taking tamoxifen and the result of ultrasound examination, the initial assessment focused more on endometrial pathology. Therefore, hysteroscopy and segmental diagnostic curettage were performed as the initial approach. The postoperative pathology report revealed diffuse glandular hyperplasia without significant atypia in the cervical curettage specimen. Immunohistochemical staining showed diffuse expression of MUC6 and partial expression of P16, suggesting gastric-type epithelial lesion. Therefore, we proceeded to perform a comprehensive pelvic magnetic resonance imaging (MRI) to further evaluate the condition. Contrary to the ultrasound results, the pelvic MRI indicated a cervical mass measuring approximately 4.9*4.3 cm, without involvement of the lower segment of the uterus or pelvic vessels, and no enlargement of the parametrium, vaginal extension, or pelvic lymph nodes.

Considering the possibility of gastric-type epithelial lesion, a second procedure of cervical conization was performed. Gross examination of the specimen revealed multiple cystic masses containing clear and mucinous fluid. Histopathological examination indicated lobular endocervical glandular hyperplasia, with focal involvement of the resection margins by the lesion glands. Immunohistochemical staining showed pyloric gland metaplasia. Staining results were as follows: MUC6 (+), CEA (partially +), P16 (patchy +), Ki-67 (+<1%). Based on the pathological findings and the patient’s individual circumstances, we recommended a laparoscopic total hysterectomy with bilateral salpingectomy, which revealed atypical lobular endocervical glandular hyperplasia on postoperative pathology examination.

Another case similar to the previous one involved a 41-year-old female who had been experiencing vaginal discharge for over 3 years. The patient sought medical attention at local hospitals multiple times, where gynecological examinations, vaginal discharge tests, HPV screening, and ultrasound examinations did not reveal any significant abnormalities. Consequently, the doctors suspected that the patient’s symptoms of vaginal discharge were caused by vaginitis and prescribed appropriate vaginal medications. However, despite the treatment, the patient’s symptoms did not improve, leading her to seek further medical care at our hospital. In 2014, the patient underwent cervical conization for high-grade squamous intraepithelial lesion (HSIL) at our hospital. Regular annual cervical cancer screenings since then have shown normal results, and the patient has not received HPV vaccination. The patient has had two cesarean sections and ten miscarriages. There is no family history of tumors or genetic diseases.

**Figure 1:**
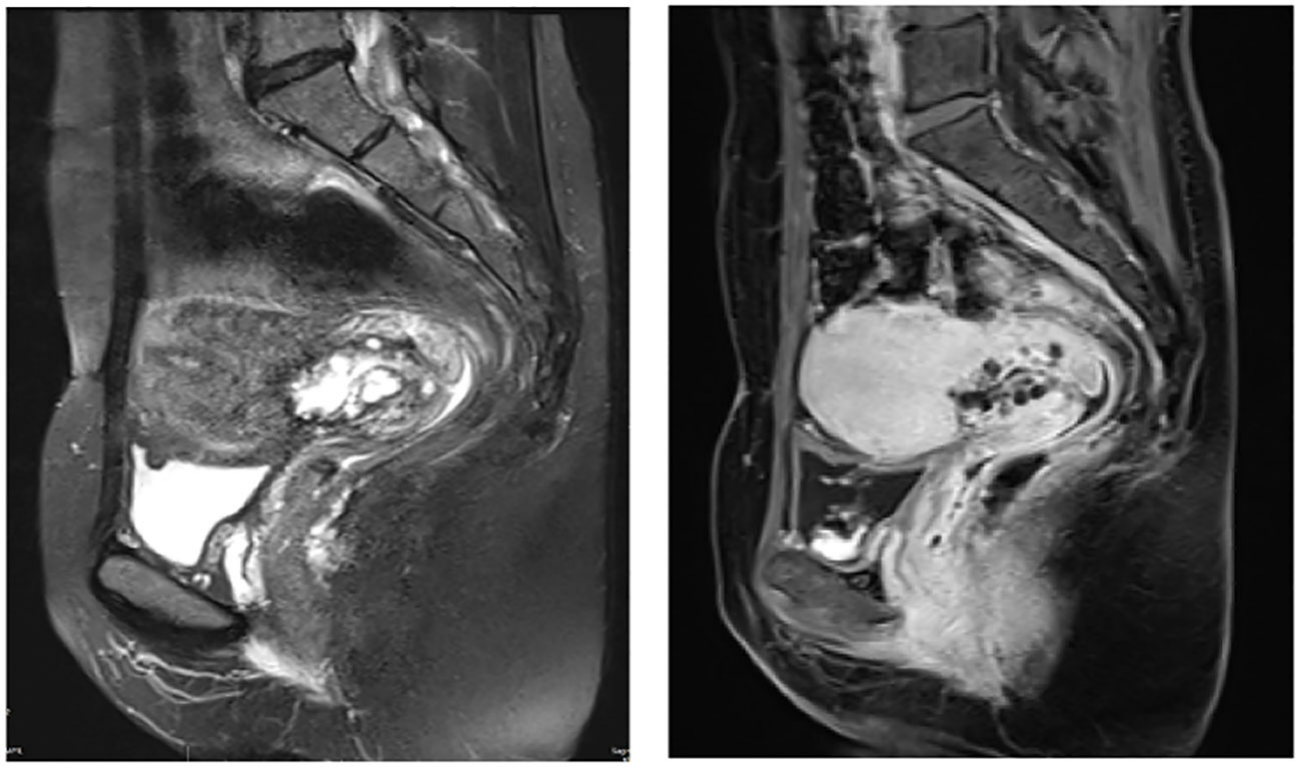
Pelvic magnetic resonance imaging (MRI) revealed presence of mass near the internal os of the upper segment of the cervix. This mass exhibited a combination of cystic and solid components, forming a distinctive pattern (case2).

Upon gynecological examinations, a small amount of thin and watery discharge was observed in the vagina. The cervix showed post-conization changes with touch bleeding. It was enlarged with a diameter of approximately 3 cm and had a slightly firm texture, without involvement of the parametrium. Pelvic MRI showed a cervical mass measuring approximately 3.7*1.8 cm, without involvement of the lower segment of the uterus or pelvic vessels, and no enlargement of the parametrium, vaginal extension, or pelvic lymph nodes (**Figure 1**).

**Figure 2:**
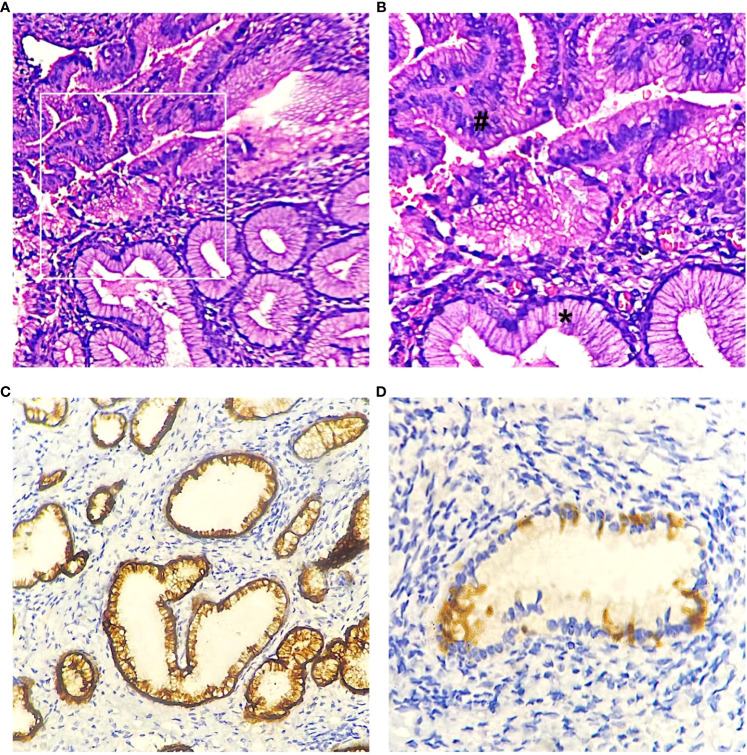
LEGH (*) and ALEGH (#) were observed in hematoxylin and eosin (HE) stained slides. **(A)** case 2, magnification x200. **(B)** case2, magnification x400. **(C)** Immunohistochemical staining revealed positive expression of MUC6 (case2). **(D)** negative or patchy positive expression of P16 (case1).

Based on the patient’s condition and previous diagnostic and therapeutic experience, we performed cervical cold knife conization, hysteroscopy, and diagnostic curettage at the same time. Intraoperatively, cervical enlargement and honeycomb-like structures on both sides of the cervical internal os were observed under hysteroscopic visualization. A cone-shaped excision of cervical tissue was performed, with a cone height of approximately 4.0 cm. The postoperative pathology report revealed atypical lobular endocervical glandular hyperplasia in the cervix, with proliferative glandular tissue at the resection margins (**Figure 2**). Immunohistochemical staining showed MUC6(+), CEA (focal+), P16 (-), Ki-67 (3%). Subsequently, the patient underwent laparoscopic total hysterectomy with bilateral salpingectomy. Due to the patient’s age of 41, both ovaries were preserved. Postoperative pathological examination revealed LEGH in the residual cervical tissue in the focal area after cervical conization, no residual ALEGH lesion was found. Both patients did not receive postoperative chemotherapy and have been followed up to date with a favorable condition.

## 3 Discussion

Cervical cancer ranks as the fourth most common type of cancer among women worldwide (6). The implementation of cervical cancer screening programs has significantly reduced the incidence of cervical squamous cell carcinoma, while the relative incidence of cervical adenocarcinoma has increased (7). Among these cases, approximately 90% of cervical adenocarcinomas are associated with high-risk HPV infections (8). GAS is the predominant subtype among cervical adenocarcinomas not related to HPV, accounting for approximately 20% to 25% of all cases (9). In the past, GAS has been categorized as a subtype of cervical mucinous adenocarcinoma, and MDA is considered a rare and well-differentiated variant of GAS (10). However, with recent research findings, it has been discovered that HPV-related adenocarcinoma and non-HPV-related adenocarcinoma have distinct molecular foundations (11). Subsequently, in the latest 2020 version of the World Health Organization (WHO) classification of cervical adenocarcinoma, GAS is classified as a subtype of non-HPV-related adenocarcinoma (12). Unlike usual HPV associated endocervical adenocarcinoma, which typically remain localized to the pelvis (while sparing ovaries) and regional lymph nodes until late in the disease course, GAS frequently metastasize to ovaries, the abdomen, the omentum, and distant sites (13). Although GAS is not common, its relative prevalence may increase with the widespread use of HPV vaccines. Additionally, HPV-based primary screening programs have a lower detection rate for precursor lesions. Therefore, it is important to pay attention to GAS in clinical practice, which necessitates further investigation into its etiology and approaches for prevention and screening.

LEGH is a rare lesion, occurring in just 0.7% of 1169 uterine samples from a single institution (14). Under the microscope, the cervical glandular tissue appears as lobular hyperplasia, with larger glandular structures in the center of the lobules and smaller to medium-sized glands densely arranged around them. The glands are lined with tall columnar mucinous epithelium, sometimes containing eosinophilic granular cytoplasm and basal nuclei (1, 14). The lining cells of the glands show no significant atypia, and mitotic figures are rare. Mikami Y et al. (15) confirmed that the proliferative glands exhibit a unique gastric phenotype (gastric metaplasia), as demonstrated by immunohistochemistry staining for HIK1083, a specific antibody for gastric pyloric glands. LEGH may be misdiagnosed as MDA, but the orderly arrangement of lobular structures with clear boundaries, lack of irregular deep stromal infiltration, and absence of stromal reactions such as increased collagen can help differentiate it.

When glandular structures and their covering epithelium show atypia, it is referred to as ALEGH. Mikami et al. (2) first described the pathological features of ALEGH, which include at least four of the following non-classical features within the lesions of LEGH: (1) nuclear enlargement, (2) irregular nuclear contour, (3) distinct nucleoli, (4) coarse chromatin texture, (5) loss of polarity, (6) occasional mitotic figures, (7) apoptotic bodies and/or nuclear debris in the lumen, (8) infolding of epithelium or distinct papillary projection with fine fibrovascular stroma. Their study also reported three cases of simple ALEGH and six cases of ALEGH with MDA. Compared to LEGH, ALEGH is even rarer, as none of the 1169 uterine specimens exhibited the atypical features despite 0.7% showing LEGH (14).

Mikami et al. (2) found that both ALEGH and MDA exhibit decreased expression of p16^INK4^, and recent studies indicate a strong correlation between diffuse staining of p16^INK4^ in the nucleus and cytoplasm and high-risk HPV-related lesions (16, 17). Currently recognized HPV-related invasive precancerous lesions, including adenocarcinoma *in situ* (AIS) and squamous intraepithelial lesion (SIL), are presently found in the transformation zone of the cervix. In contrast, LEGH often affects the endocervical canal away from the transformation zone (18). Therefore, scholars speculate that the decreased expression of p16^INK4^ in LEGH and LEGH-derived MDA is associated with the absence of high-risk HPV involvement in tumor development. To further elucidate the correlation between LEGH and MDA, Kawauchi et al. (5) conducted a related study using molecular genetics and immunohistochemistry. Genomic hybridization revealed that among the 14 LEGH cases analyzed, three exhibited chromosomal imbalances similar to MDA, specifically an increase in chromosome 3q and loss of chromosome 1p. The chromosomally imbalanced LEGHs showed varying degrees of atypia in the proliferative glandular epithelium.

LEGH can occur in patients with Peutz-Jeghers syndrome (PJS) with STK11 mutations (19), and in the sporadic cases, the most common mutations are GNAS (42%), KRAS (5%), and STK11 (10%) (20). PJS is associated with various tumors that affect the female reproductive system in the field of gynecologic oncology. This category includes GAS of the cervix, mucinous neoplasms of the ovaries, and a distinct variant known as sex cord tumor with annular tubules (SCTAT) of the ovaries. Additionally, there are sporadic connections with other tumors of the ovary’s sex cord-stromal (8, 21, 22). Research estimates suggest that the prevalence of GAS in individuals with PJS varies between 15% and 30%. Furthermore, studies have revealed that the typical age at which PJS patients are diagnosed with GAS is 33 years, indicating that GAS tends to manifest at a relatively early age in individuals with PJS. Further examination of the data indicates that approximately 10% of GAS cases are associated with PJS (23). Therefore, it is recommended that when diagnosing GAS, the pathology report should include a remark acknowledging the association with PJS (24). Moreover, synchronous mucinous growths may potentially be associated with gastric-type glandular growths in different parts of the female reproductive system, such as mucinous transformation and abnormal cell growth in the endometrium, fallopian tubes, and ovaries. Additionally, it may also be related to cervical LEGH or MDA/GAS (15, 25).

LEGH commonly occurs in women aged 45-48 years and is typically incidentally found in specimens from hysterectomy or cervical conization procedures (1, 18). The two cases of ALEGH reported in our study were aged 41 and 47, both falling within the high-risk age range for LEGH. Patients with LEGH may present with watery vaginal discharge and/or yellow-orange mucin within the cytoplasm of glandular cells on cytological smears (9, 26). Therefore, clinicians should pay attention to patient complaints. ALEGH needs to be differentiated from other conditions causing vaginal discharge, such as urinary incontinence, vaginitis, and endometrial lesions. Additionally, emphasis should be placed on gynecological examinations. In our two cases, we observed increased watery discharge on the cervical surface during direct visualization, which was more pronounced under magnification with colposcopy, while no obvious lesions were visible on the cervical surface. This is because ALEGH often affects the upper segment of the cervical canal and is frequently limited to the inner half of the cervical canal wall. Radiologically, LEGH typically appears as a well-defined mass with a combination of cystic and solid components, exhibiting a “cosmic” pattern on MR imaging (24). In Takatsu et al.’s (27) study of 39 LEGH cases, this pattern was observed in 36 cases (87%). Similarly, this pattern was observed in our two reported cases. Based on the characteristics of ALEGH, it can be differentiated from other high-grade cervical intraepithelial lesions, including AIS and HSIL. HSIL is more commonly found in the transformation zone of the cervix, and abnormal findings can be detected through cytological screening and HPV testing. Characteristic images observed during colposcopy can also assist in the differentiation (28). In contrast to HSIL, AIS is more likely to occur within the cervical canal, with lesions scattered and distributed in a skip pattern. Cytological sampling limitations may lead to missed diagnose, however, most AIS are associated with HPV infection, especially type 18 (29). Therefore, a differential diagnosis can be made by combing patient symptoms, HPV and cytological screening results, colposcopy examination, and characteristic imaging findings.

Due to the growing recognition of this condition, numerous researchers have investigated various methods to identify gastric-type cervical lesions. In Japan, a latex agglutination test utilizing HIK1083 has been developed and is commercially accessible. This test aims to identify gastric mucin in cervical secretions, offering a potential screening method for these lesions. Omori et al. (30) demonstrated that this test has outstanding sensitivity and specificity in identifying gastric-type lesions in the cervix. Out of the 44 women who experienced heightened vaginal secretions, displayed yellow mucinous glandular cells on cervical smears, and/or had cystic cervical findings on imaging, the latex agglutination test yielded positive results for all 26 women. These women were diagnosed with gastric-type cervical glandular lesions, including LEGH, atypical LEGH, and MDA/GAS, as confirmed by histological examination. All 31 asymptomatic control patients tested negative, and none of the 18 patients who exhibited these symptoms or indications but had negative results showed gastric-type lesions. The latex agglutination test using HIK1083 may be helpful in detecting MDA or GAS, but it should be used in conjunction with other methods for distinguishing between benign and malignant lesions. According to the research of Takatsu (27), LEGH can be strongly suggested when MRI findings display both cystic and solid components in a cosmic pattern, absence of significant glandular atypia on cytological smears, and a highly positive gastric mucin test. On the other hand, the presence of widespread solid alterations on MRI and the identification of unusual glandular cells on smears indicate the possibility of MDA or GAS. Therefore, the combination of MRI, Papanicolaou smears, and/or latex agglutination tests can enhance the accuracy of predictions compared to separate examinations. Histologically, GAS exhibits a diverse spectrum of lesions. MDA appears as a well-differentiated mucinous adenocarcinoma under the microscope. It is composed of irregularly shaped “claw-like” glands of varying sizes, infiltrating in an irregular pattern and often extending deep into the layers of the cervix (21). The main histopathological differentiating factor is the depth of malignant tumor infiltration, which exceeds that of normal glands or approaches thick-walled blood vessels, with possible involvement of blood vessels and nerves (13, 31). However, in superficial biopsies and cone specimens, it may be difficult to effectively distinguish from ALEGH, and in such cases, immunohistochemistry markers such as P16, Ki67, P53, PAX2, and CEA may be used for additional differentiation (32). In individual cases, uterine resection may be necessary to further clarify the presence of deep infiltration.

The diagnosis of ALEGH requires pathological evidence, but obtaining biopsy samples can be relatively challenging. Cone biopsy of the cervix is a common method for obtaining biopsy tissue of cervical lesions (33). However, due to the different sites where the lesions commonly occur, ALEGH requires deeper excision of the cervix compared to CIN. This increases the risk of postoperative pregnancy complications such as miscarriage or preterm birth due to cervical shortening, which affects the fertility of women of childbearing age (34–36). Additionally, it also increases the risk of cervical stenosis or occlusion, which can impact the patient’s menstrual status and quality of life (34). Researchers have attempted hysteroscopic biopsy in 13 patients with MRI findings suggestive of potential LEGH or malignant tumors. The results suggest that hysteroscopic biopsy can provide targeted excision of the cervix while maintaining diagnostic accuracy (37). However, the number of cases studied is limited, and more data are needed to support these findings.

In our first reported case, the outpatient hysteroscopic surgery did not yield sufficient diagnostic tissue specimens, with only a small amount of cervical scraping suggesting gastric-type glandular epithelial lesions. This also indicates the relative difficulty in obtaining specimens during hysteroscopy. Subsequent cone biopsy tissue was still insufficient for a diagnosis of ALEGH, which may be due to inadequate depth of the cone biopsy specimen. Therefore, preoperative evaluation is crucial. In the second case, due to preoperative ultrasound and MRI findings of a mass in the upper part of the cervix, we considered the possibility of GAS/MDA and its precancerous lesions. We performed a combination of cone biopsy, hysteroscopy, and diagnostic curettage. Under hysteroscopic visualization, we observed honeycomb-like structures on both sides of the cervical internal os and obtained sufficient tissue specimens for a definitive diagnosis, thus improving the diagnostic efficiency. Therefore, further clinical studies are needed to explore the most appropriate biopsy methods for ALEGH/LEGH.

There is currently no unified standard for the management of ALEGH in clinical practice. Most scholars believe that a reasonable choice is hysterectomy (9), and pathologists should perform multi-section examination of the tissue to exclude possible coexisting invasive cancer and avoid misdiagnosis. The 5-year survival of patients with LEGH and ALEGH who underwent hysterectomy was 100%, in contrast to only 54% for the invasive adenocarcinomas (38). Cervical conization is considered relatively unreasonable because the lesion is located higher, and the surgical margins are usually positive. Only for those who have fertility requirements or are unwilling to remove the uterus. No ALEGH or more severe lesions were observed, and for those with negative margins, they will undergo uterus removal after childbirth or be actively followed up. Relevant literature reports that MRI (cystic lesion area enlargement, solid area or invasive boundary) combined with cervical cytology (atypical glandular cell lesion degree increase) changes suggest the malignant transformation of cervical polycystic lesions (39), which can be used as a management plan for those who are willing to retain the uterus. However, in the treatment of the first case, despite the histopathological findings of the biopsy tissue suggesting LEGH without considering atypical lesions, considering the patient’s history of breast cancer and age, and after consultation with the patient and family, we still recommended hysterectomy, and the final excised tissue confirmed ALEGH. In the second case, as the biopsy tissue indicated ALEGH, we directly recommended hysterectomy for the patient.

## 4 Conclusion

As a precancerous lesion of GAS/MDA, ALEGH exhibits certain overlaps in clinical manifestations, cell morphology, and immunophenotype. It is crucial for clinicians and pathologists to enhance their understanding of this disease and strengthen the management of patients with cervical polycystic lesions. Even in cases where cervical cytology screening and HPV detection yield negative results for patients with long-term vaginal discharge, the possibility of cervical precancerous lesions should still be considered. This is particularly important for patients with characteristic imaging manifestations, as they need to remain highly vigilant for ALEGH. When obtaining histological specimens, it is advisable to choose the appropriate method based on the patient’s condition and lesion location. Hysteroscopy and conical resection can be considered, especially to ensure the depth of the material. Additionally, a combination of characteristic immunohistochemistry and special staining can aid in early diagnosis.

## Data availability statement

The raw data supporting the conclusions of this article will be made available by the authors, without undue reservation.

## Ethics statement

The studies involving humans were approved by Hunan Provincial Maternal and Child Health Care Hospital. The studies were conducted in accordance with the local legislation and institutional requirements. Written informed consent for participation in this study was provided by the participants’ legal guardians/next of kin. Written informed consent was obtained from the individual(s) for the publication of any potentially identifiable images or data included in this article.

## Author contributions

ZW: Writing – original draft, Writing – review & editing. SL: Writing – original draft, Writing – review & editing. NS: Data curation, Formal analysis, Writing – review & editing. YT: Resources, Supervision, Validation, Visualization, Writing – review & editing. PW: Resources, Supervision, Validation, Visualization, Writing – review & editing. PZ: Resources, Supervision, Validation, Visualization, Writing – review & editing. CS: Resources, Supervision, Validation, Visualization, Writing – review & editing.
